# Incidental crossed fused renal ectopia detected during prostate cancer staging: A photorealistic three‐dimensional rendering

**DOI:** 10.1002/bco2.70039

**Published:** 2025-06-02

**Authors:** Kei Ushijima, Kosuke Kojo, Tomoyuki Ohta, Keita Okamoto, Daisuke Numahata, Hiromu Inai, Katsunori Uchida, Hideki Takeshita, Hiroyuki Nishiyama, Tatsuya Takayama

**Affiliations:** ^1^ Department of Urology International University of Health and Welfare Hospital Nasushiobara Japan; ^2^ Center for Human Reproduction International University of Health and Welfare Hospital Nasushiobara Japan; ^3^ Department of Urology, Institute of Medicine University of Tsukuba Tsukuba Japan; ^4^ Tsukuba Clinical Research & Development Organization University of Tsukuba Tsukuba Japan; ^5^ Department of Radiology International University of Health and Welfare Hospital Nasushiobara Japan

Crossed fused renal ectopia (CFRE), also referred to as crossed fused ectopic kidney, is a congenital malformation in which both kidneys are located unilaterally, and one ureter opens into the ureteral orifice on the contralateral side.^1^ In addition to cases detected in infancy due to multiple congenital anomalies or in adolescence due to delayed menarche associated with concurrent genital malformations, CFRE is often incidentally discovered in adults without significant complications.^2^ An autopsy series reported an incidence of 1 in 2000–7500 cases, while a large CT study estimated an occurrence rate of approximately 1 in 3078 scans, making CFRE the second most common fusion anomaly after horseshoe kidneys.^1^

However, the clinical significance of asymptomatic CFRE in older adults is unclear. Notably, only three published cases have reported CFRE in patients undergoing evaluation for prostate cancer.^3^, ^4^, ^5^ Herein, we present the fourth case discovered incidentally in a 72‐year‐old man with no remarkable medical history. He underwent a prostate biopsy to confirm prostate cancer, and a subsequent CT scan for metastatic screening revealed a CFRE. With his written informed consent, we reconstructed a photorealistic three‐dimensional (3D) image from the CT data for illustrative purposes.

This imaging study was conducted as a part of an ongoing multi‐institutional observational research project aimed at visualising various genitourinary malformations (University of Tsukuba, Institutional Review Board approval number: R05‐199). Portal‐phase contrast‐enhanced CT images (2‐mm slice thickness) were acquired for prostate cancer staging. We performed image segmentation and reconstruction using SYNAPSE 3D Version 7.00 (Fujifilm Medical Co., Ltd., Tokyo, Japan), also known as SYNAPSE VINCENT in Japan, following a previously described manual approach.^6^ We meticulously segmented the renal parenchyma, associated arteries and veins (including bilateral gonadal veins), ureters, bilateral adrenal glands and bones. For final rendering, we employed a technique commonly known as cinematic rendering, provided as ‘Photorealistic Rendering’ in SYNAPSE 3D. Compared with standard volume rendering, cinematic rendering offers enhanced shadow realism and depth perception, producing ‘photo‐like’ images that could potentially improve anatomical education for students, enhance communication with patients and help clinicians plan surgical interventions by providing images that more closely resemble the intraoperative view.^7^

Figure 1 presents the final rendering clearly demonstrating the inferior location of the ectopic kidney relative to the orthotopic kidney. Both renal pelves were oriented in the same direction, classically described by McDonald and McClellan as ‘inferior ectopia’, the most common subtype of CFRE.^8^ Importantly, the image also elucidated the arterial and venous supply to both the ectopic and orthotopic kidneys and showed that the left adrenal gland is situated above the fused kidneys, whereas the right adrenal gland remained in the right renal fossa, both without apparent anomalies. Understanding the precise vascular anatomy is crucial for surgical planning, particularly if nephron‐sparing interventions are necessary for renal tumours.^9^ Although few studies have explicitly documented the status of adrenal glands in CFRE, one contrasting case reported complete absence of one adrenal gland in the ectopic kidney.^10^ Therefore, our case provides additional insights into the variable anatomical presentation of this rare yet significant malformation.

**FIGURE 1:**
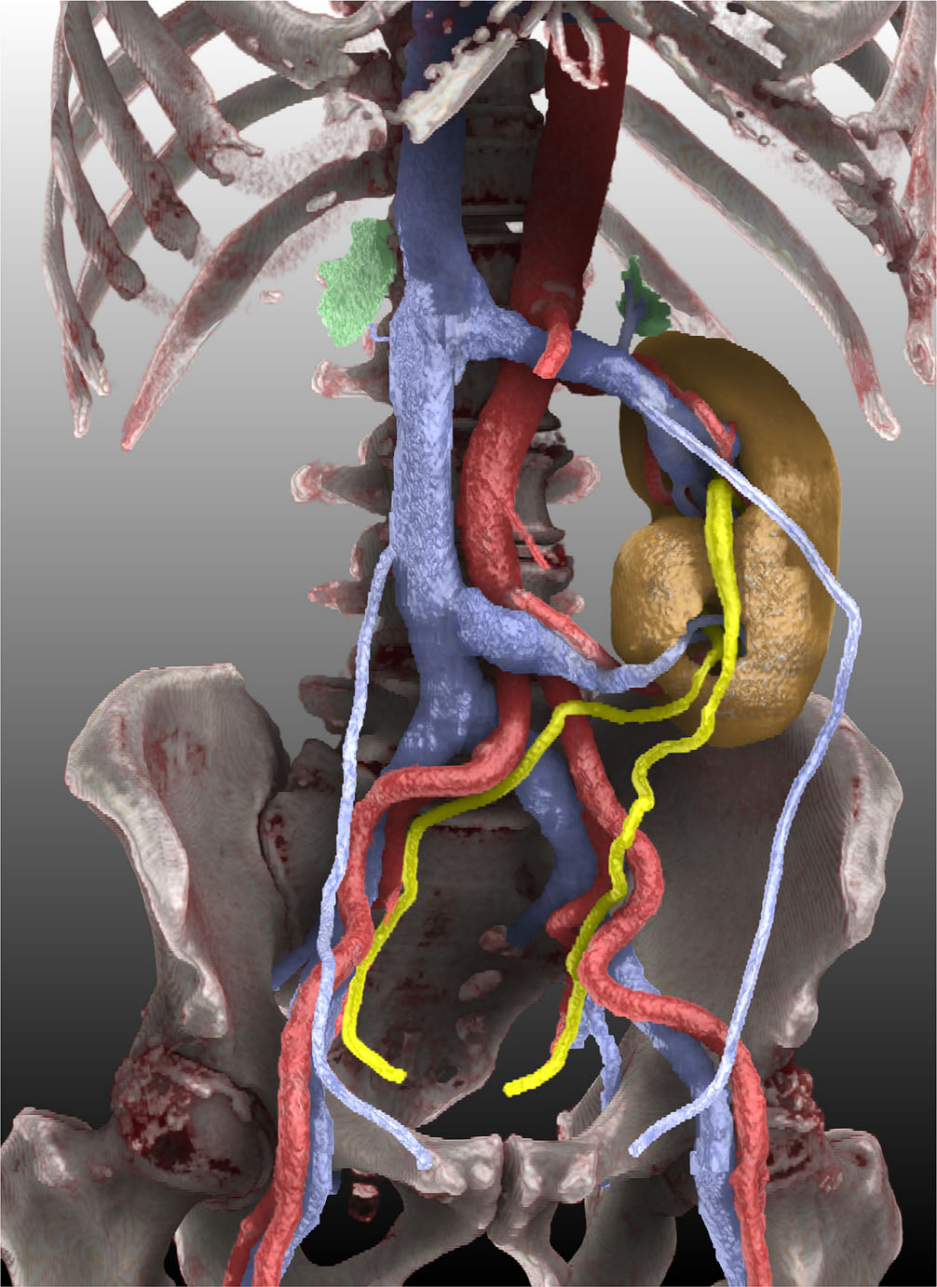
Cinematic rendering of a crossed fused renal ectopia and surrounding organs, incidentally discovered on a CT scan performed for metastatic screening in a 72‐year‐old man with no significant past medical history and newly diagnosed prostate cancer. The fused renal parenchyma is depicted in ocher, arteries in red, veins (including bilateral gonadal veins) in blue, both ureters in lemon yellow, bilateral adrenal glands in light green and bones in white and reddish brown, respectively.

## AUTHOR CONTRIBUTIONS

Kei Ushijima and Kosuke Kojo wrote the original manuscript draft. Kosuke Kojo and Tomoyuki Ohta created images using cinematic rendering. Keita Okamoto, Daisuke Numahata, Hiromu Inai, Katsunori Uchida, Hideki Takeshita, Hiroyuki Nishiyama and Tatsuya Takayama critically revised the manuscript.

## CONFLICT OF INTEREST STATEMENT

The authors declare no conflicts of interest.
